# Analysis of Immune Checkpoint Drug Targets and Tumor Proteotypes in Non-Small Cell Lung Cancer

**DOI:** 10.1038/s41598-020-66902-0

**Published:** 2020-06-17

**Authors:** Daniel C. Liebler, Timothy R. Holzer, Alexander Haragan, Ryan D. Morrison, Leslie O’Neill Reising, Bradley L. Ackermann, Jeff A. Fill, Andrew E. Schade, Aaron M. Gruver

**Affiliations:** 1 Protypia, Inc, Nashville, TN USA; 2 Lilly Research Laboratories, Eli Lilly and Company, Indianapolis, IN USA; 3 Institute of Translational Medicine, University of Liverpool, Liverpool, UK

**Keywords:** Non-small-cell lung cancer, Cancer immunotherapy, Mass spectrometry, Proteomics

## Abstract

New therapeutics targeting immune checkpoint proteins have significantly advanced treatment of non-small cell lung cancer (NSCLC), but protein level quantitation of drug targets presents a critical problem. We used multiplexed, targeted mass spectrometry (MS) to quantify immunotherapy target proteins PD-1, PD-L1, PD-L2, IDO1, LAG3, TIM3, ICOSLG, VISTA, GITR, and CD40 in formalin-fixed, paraffin-embedded (FFPE) NSCLC specimens. Immunohistochemistry (IHC) and MS measurements for PD-L1 were weakly correlated, but IHC did not distinguish protein abundance differences detected by MS. PD-L2 abundance exceeded PD-L1 in over half the specimens and the drug target proteins all displayed different abundance patterns. mRNA correlated with protein abundance only for PD-1, PD-L1, and IDO1 and tumor mutation burden did not predict abundance of any protein targets. Global proteome analyses identified distinct proteotypes associated with high PD-L1-expressing and high IDO1-expressing NSCLC. MS quantification of multiple drug targets and tissue proteotypes can improve clinical evaluation of immunotherapies for NSCLC.

## Introduction

New drugs directed at immune checkpoint proteins and their co-regulators have dramatically advanced oncology therapeutics. Antibody inhibitors of PD-1 (PDCD1) and PD-L1 (CD274) have become first-line therapeutics for a growing list of cancers, including non-small cell lung cancer (NSCLC)^1–6^. Despite dramatic, durable responses in some patients, approximately 36–55% of NSCLC patients without driver alterations fail to respond to either immune checkpoint monotherapies or combinations with chemotherapy^7^. This challenge has led to exploration of combination therapies directed against other immune checkpoints and other oncology targets^8,9^.

However, the large number of validated drug targets and the complexity of their interactions presents a substantial challenge in systematic development of new therapeutics and therapeutic combinations. In NSCLC, clinical trials are evaluating drugs targeting multiple immune checkpoint proteins PD-1, PD-L1, LAG3, TIM3 (HAVCR2), VISTA (VSIR), CD40, and GITR (TNFRSF18), as well as combinations of immune checkpoint inhibitors and other targets. For example, multiple trials are evaluating the PD-1 inhibitor pembrolizumab in combination with antibodies against LAG3 (NCT03516981; NCT02720068), RORC (NCT03396497), CTLA4 (NCT03516981), with small molecule inhibitors of JAK1 (NCT03425006), EGFR (NCT02364609), MEK1/2 (NCT03299088), PIK3CD (NCT03257722) and IDO1 (NCT03343613; NCT03322540), and with vaccines targeting MUC1/CEA (NCT02840994) or patient-specific tumor antigens (NCT03380871). The success of combination therapies will depend in large part on understanding the relationships between drug target protein abundances and tumor phenotypes. Quantitative verification of drug targets in tumor tissues is an underappreciated and unmet need in therapeutic development.

Analysis of drug protein targets in tissues has been done largely by immunochemical methods, particularly immunohistochemistry (IHC)^10^. IHC was developed as a qualitative tool to facilitate interpretation of H&E stained specimens and its greatest utility is in capturing the spatial relationships between protein expression and cell or tissue morphology. However, the application of IHC as a quantitative protein measurement technique is constrained by limitations of the platform. IHC assays based on chromogenic detection display limited dynamic range and multiplexing capacity^11^. The number of proteins that can be detected in a single tissue section by IHC is limited, even with the use of multiplexed staining approaches. Lack of availability of specific antibodies precludes analysis of many proteins by IHC. Finally, IHC antibody detection of some proteins, such as PD-L1 declines with time during slide storage^12,13^.

Analysis of mRNA transcripts is widely used as an indicator of gene expression and mRNA expression signatures themselves have established utility as diagnostics^14^. However, mRNA expression is not necessarily a reliable surrogate for protein abundance. Combined proteomic/genomic analyses demonstrate that mRNA levels do not predict abundance of the corresponding proteins across cohorts of colon, ovarian and breast tumor tissues^15–18^. mRNA/protein correlations vary dramatically with gene functions and ontologies and comparison of mRNA and protein co-expression networks in these studies revealed that protein co-expression networks display greater functional coherence, defined as fidelity to annotated gene ontologies shared by co-expressed mRNAs or proteins^19^.

New mass spectrometry (MS) platforms enable deep quantitative analysis of cell and tissue proteomes. The integration of a proteome data layer with genomic, transcriptomic and other omic data enables proteogenomics, which affords a comprehensive view of the translation of biological information from gene to phenotype^20,21^. Global MS analysis generates a large inventory of identified proteins and enables quantitative estimates of differences in protein abundances across multiple samples. Targeted MS analysis affords more sensitive and precise measurement of a specific set of protein targets. Of particular importance for the analysis of clinical tissue specimens is the ability of MS to analyze proteins from formalin-fixed paraffin-embedded (FFPE) tissue specimens^22,23^. A key limitation of MS methods for drug target quantitation is that sample processing does not preserve protein localization in tissue components. Moreover, due to the relatively recent emergence of protein MS platforms, the clinical impact of MS-based protein biomarkers remains largely untested.

We combined targeted MS of immune checkpoint proteins and global MS to analyze FFPE specimens from a set of 46 NSCLCs, which we also analyzed by RNA-Seq, DNA sequencing to analyze tumor mutation burden (TMB) and IHC. The targeted assays measured PD-1, PD-L1, PD-L2 (PDCD1LG2), IDO1, LAG3, TIM3, ICOSLG, VISTA, GITR and CD40. Comparison of the datasets demonstrates the unique value of MS measurements for protein level analysis of drug targets and their associated proteotypes.

## Results

### Targeted MS, mRNA, IHC and TMB analysis of PD-1, PD-L1 and IDO1 in 46 NSCLC FFPE specimens

**Figure 1:**
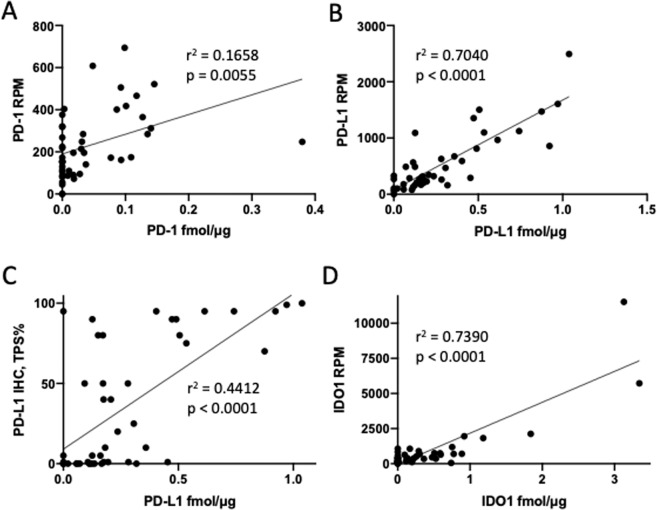
Correlation of MS protein with mRNA expression for PD-1 (**A**), PD-L1 (**B**) and IDO1 (**D**) in the 46 sample NSCLC cohort. MS protein is measured in fmol/mg tissue protein, whereas mRNA expression is expressed as reads per million mapped reads (RPM). Panel C depicts correlation of PD-L1 MS protein with TPS scoring for PD-L1 IHC with the 22C3 antibody.

We used parallel reaction monitoring (PRM) MS to perform targeted quantitative analysis of PD-1, PD-L1 and IDO1 in FFPE sections from 46 NSCLC specimens, for which we also generated RNA-Seq data (Table S1; Supplementary Dataset 1). PD-1 protein was detected by MS in 27 of the 46 tumors analyzed and mRNA was measured in 43 tumors. PD-1 protein and mRNA were weakly correlated (r^2^ = 0.1658; p = 0.0055) (Fig. 1A). PD-L1 protein was detected in 40 tumors and was correlated with mRNA abundance (r^2^ = 0.7040; p < 0.0001) (Fig. 1B). PD-L1 protein measurements by MS and IHC (measured as Tumor Proportion Score (TPS)) were weakly, but significantly correlated across the dataset (r^2^ = 0.4412; p < 0.0001) (Fig. 1C; Table S2). Tumors with > 50% PD-L1 TPS—a cutoff for clinical use of pembrolizumab—contained PD-L1 across the entire range of abundances measured by MS. At MS protein abundances between 0.2 and 0.5 fmol/μg, TPS yielded values ranging from zero to 95%. IDO1 was detected in 26 tumors and was correlated with mRNA abundance (r^2^ = 0.7390; p < 0.0001) (Fig. 1D). TMB, as measured in mutations/Mb varied by approximately 200-fold (Table S3), but was not correlated with abundance of PD-1, PD-L1 or IDO1 proteins (Fig. S1).

### Targeted MS, mRNA and TMB analysis of PD-L2, LAG3, TIM3, ICOSLG, VISTA, GITR, and CD40 in 20 NSCLC FFPE specimens

**Figure 2:**
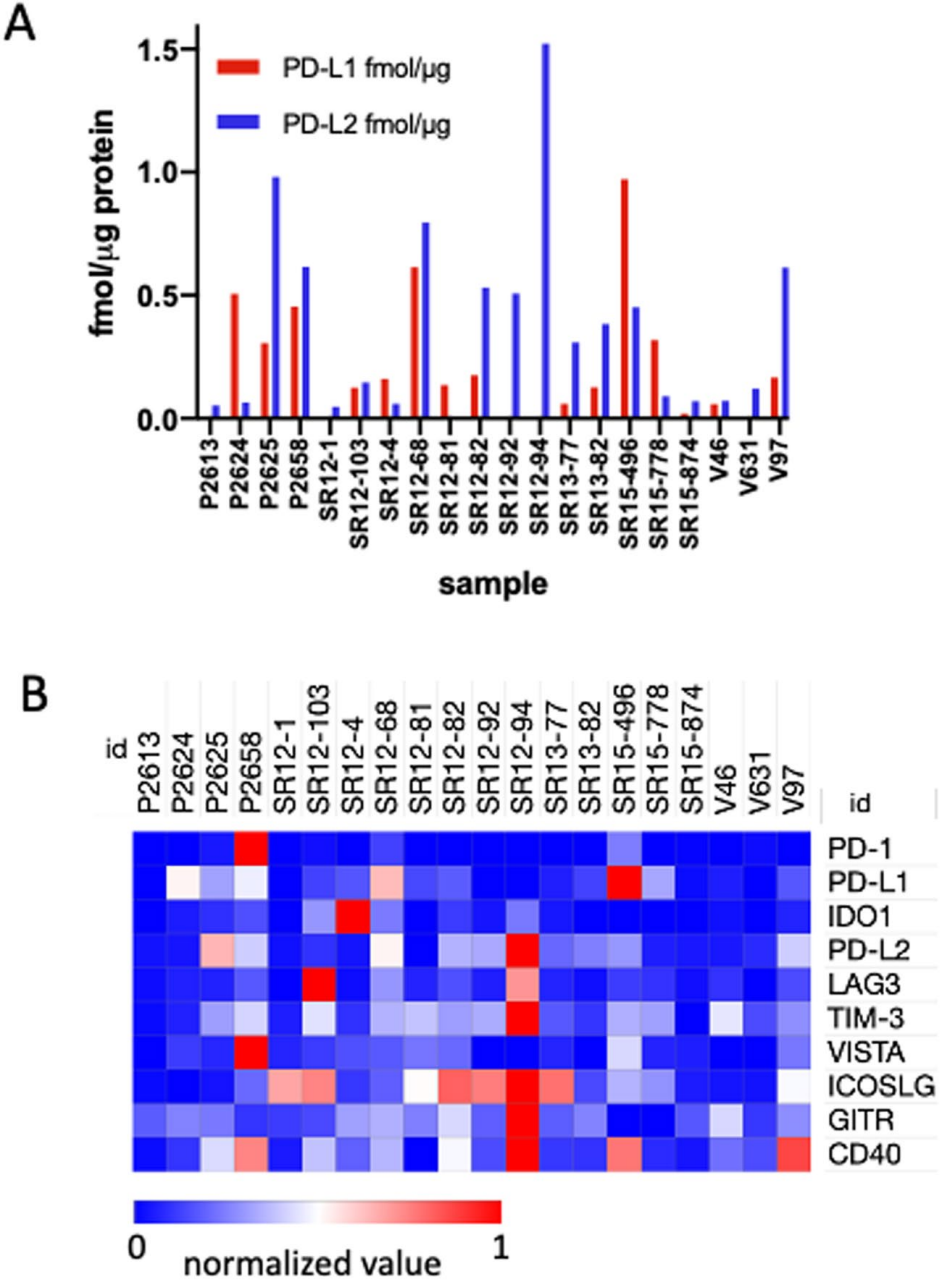
(**A**) Abundance of PD-L1 and PD-L2 MS protein in the 20 sample NSCLC subcohort. (**B**) Heatmap representation of relative abundance of PD-1, PD-L1, IDO1, PD-L2, LAG3, TIM3, ICOSLG, GITR, VISTA and CD40 in the 20 sample NSCLC subcohort. Values are normalized within rows as indicated by the color scale. Measured values are reported in Tables S1 and S4.

We analyzed a 20-specimen sub-cohort for six additional immune checkpoint proteins by targeted MS and RNA-Seq (Table S4). PD-L2 was detected in 19 of 20 tumors of this sub-cohort and was not correlated with PD-L2 mRNA abundance (r^2^ = 0.0478; p = 0.1541) (Fig. S2). PD-L2 and PD-L1 protein were co-expressed in 14 tumors and PD-L2 abundance exceeded that of PD-L1 in 15 tumors (Fig. 2A). LAG3, TIM3, ICOSLG, VISTA, GITR and CD40 were all detected in a majority of the 20 tumor subcohort over an abundance range between 10- and 40-fold (Fig. 2B; Table S4). Protein abundance was not significantly correlated with mRNA abundance for any of these proteins (Fig. S2). TMB varied by approximately 200-fold in the 20-tumor sub-cohort (Table S3) and was not correlated with abundance of any of these proteins (Fig. S1).

### Global proteome analysis of 26 NSCLC FFPE specimens

We performed a global proteomic analysis of a 26-tumor subcohort, of which 13 were squamous cell carcinomas, 11 were adenocarcinomas, and one each were described as “carcinoma” (unclassified) or “pleomorphic carcinoma” (Table S5). The global analysis yielded 1.28 M filtered MS/MS spectra mapping to 68,301 distinct peptides at a maximum Q value of 0.01. With a threshold of two distinct peptide identifications per protein, the data identified 12,301 protein groups at a protein false discovery rate of 3.43%. Spectral count data are presented in Table S6. Because we had performed targeted MS measurements for PD-L1 and IDO1 in all 26 samples, we asked how global proteotypes intrinsically differed in immune checkpoint inhibitor naïve NSCLC as a function of expression of these two drug targets.

**Figure 3:**
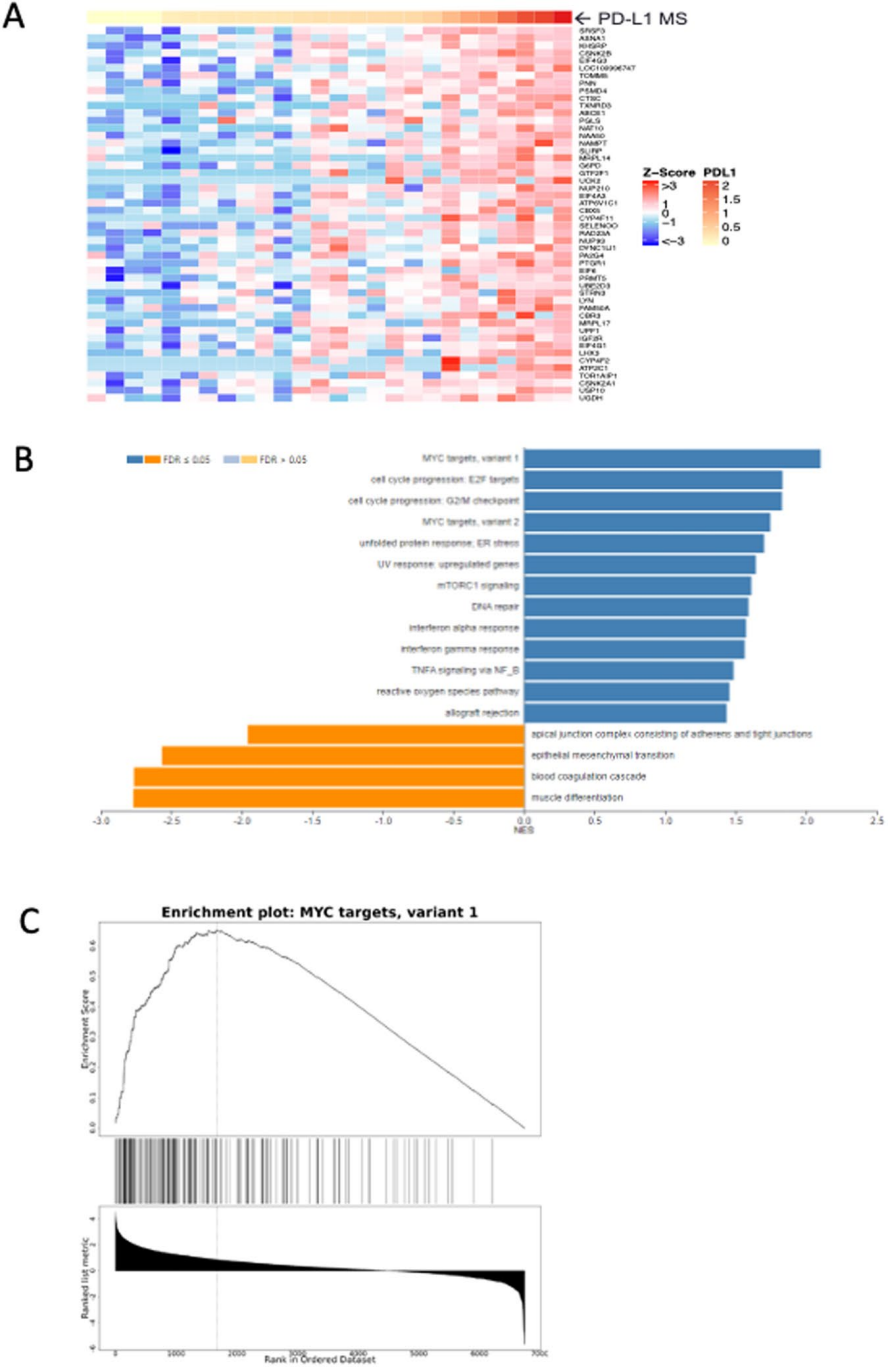
Global proteotype associated with high PD-L1 abundance in 26 sample NSCLC subcohort. (**A**) Highest abundance proteins in NSCLC global analyses as a function of PD-L1 MS protein level. (**B**) Significantly enriched Hallmark 50 pathways associated with PD-L1 MS protein level by GSEA. (**C**) Enrichment plot for Myc targets (variant 1) depicting enrichment score (upper panel) versus ranked abundance (lower panel) for all pathway proteins (depicted as tick marks) quantified in the global dataset.

Sorting of the NSCLC samples revealed global protein abundance differences based on MS PD-L1 abundance (Fig. 3A). Application of gene set enrichment analysis (GSEA) identified multiple hallmark gene sets from the Molecular Signatures Database (MSigDB) (http://software.broadinstitute.org/gsea/msigdb/collections.jsp) with significant enrichment for proteins at higher or lower abundance in concert with PD-L1 level (Fig. 3B; Supplementary Dataset 2). Among pathways most significantly enriched for proteins with elevated abundance in higher PD-L1 expressing NSCLC are Myc targets (variant 1) (Fig. 3C), Myc targets (variant 2), G2M checkpoint and E2F targets. Proteins enriched for Myc variants 1 and 2 and E2F targets include diverse participants in transcriptional regulation, RNA splicing, processing and transport, ribonucleoproteins, RNA helicases, translational regulators, ribosome and proteasome components.

**Figure 4:**
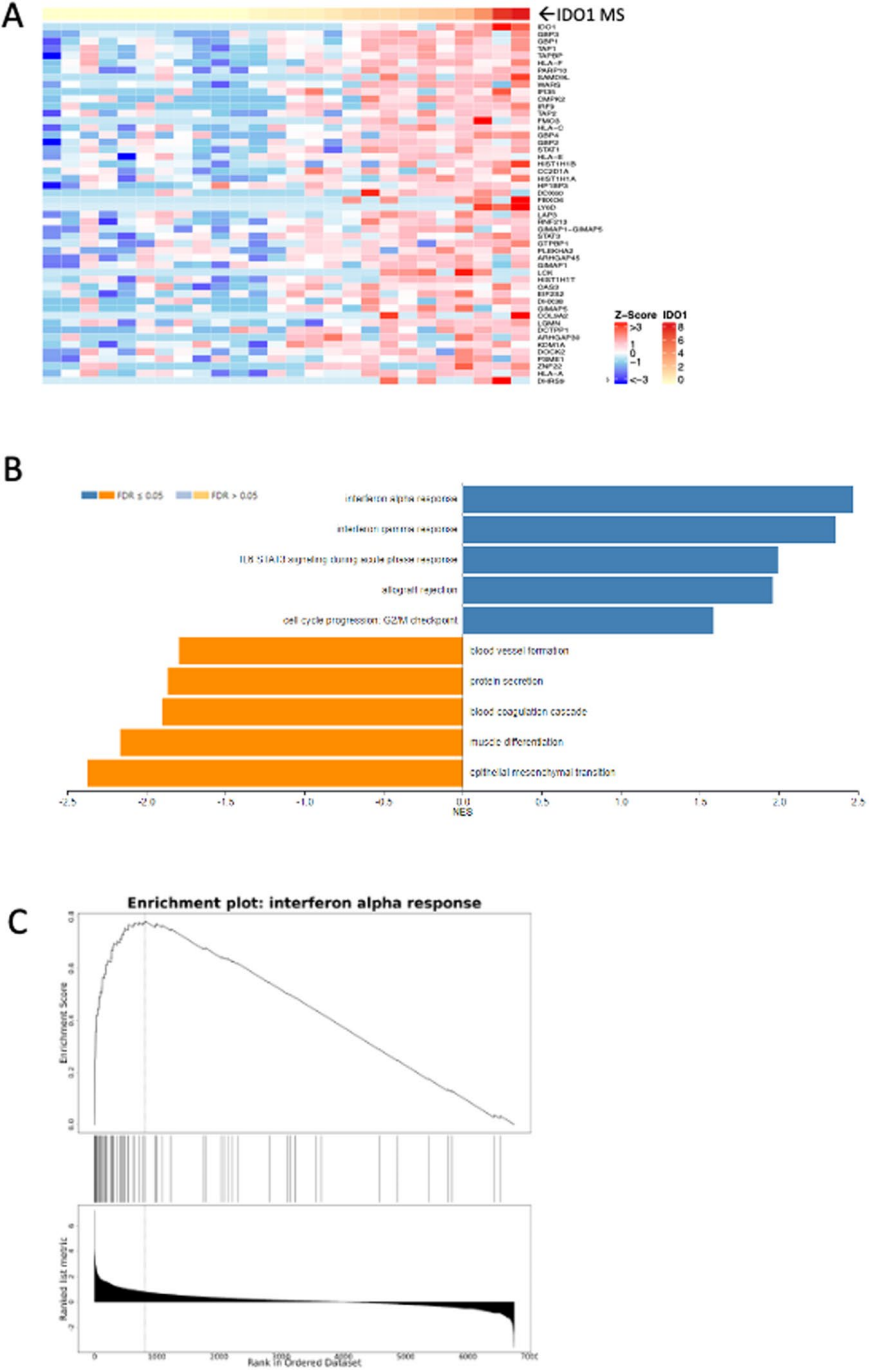
Global proteotype associated with high IDO1 abundance in 26 sample NSCLC subcohort. (**A**) Highest abundance proteins in NSCLC global analyses as a function of IDO1 MS protein level. (**B**) Significantly enriched Hallmark 50 pathways associated with IDO1 MS protein level by GSEA. (**C**) Enrichment plot for interferon alpha response depicting enrichment score (upper panel) versus ranked abundance (lower panel) for all pathway proteins quantified in the global dataset.

Sorting of the same global proteome dataset based on MS IDO1 abundance identified an entirely different set of proteotypes (Fig. 4; Supplementary Dataset 3). Pathways most significantly enriched in high IDO1 expressing NSCLC include interferon alpha- and gamma-responses, allograft rejection and IL6 STAT3 signaling during acute phase response. This group of pathways collectively include the MHC1-antigen processing proteins TAP1, TAP2 and TAPBP, as well as MHC1 component proteins HLA-A, HLA-B, HLA-C, HLA-G and B2M. These pathways also share the transcriptional regulator IRF9 and the tryptophan tRNA synthetase WARS.

## Discussion

The diversity of molecular targets for immunotherapies in NSCLC presents an opportunity to develop new therapeutic combinations, which would target key regulatory proteins with complementary roles at the tumor-immune interface. Susceptibility to a drug combination would require co-expression of protein targets at levels where inhibition by both drugs would functionally impact the tumor-immune system^24^. Drug target abundance measurements thus are critical to the success of this strategy. Here we demonstrate that targeted MS quantifies multiple immunotherapy drug targets simultaneously in archival FFPE samples. We also demonstrate that precise targeted measurements of immune checkpoint drug targets can be combined with global proteotypes to identify target-associated pathways, which provide insight into the underlying biology associated with expression of specific drug targets.

The most widely used immune checkpoint measurement is IHC analysis of PD-L1. We used the FDA-approved PD-L1 IHC 22C3 pharmDx assay to perform PD-L1 IHC analysis of the full 46 sample cohort and compared these data with MS measurements (Fig. 1C). Although IHC and MS PD-L1 measurements were correlated overall, they were strongly discordant across the middle of the MS abundance range. For MS protein values between 0.2 and 0.5 fmol/μg protein, IHC TPS values ranged from 0–95%. This result underscores fundamental differences between IHC and MS measurements. Whereas MS measures a molar quantity of protein, TPS represents a percentage of tumor cells observed to have membranous staining intensity visible at 20x or lower power magnification. Cells with very weak, barely visible staining are scored equally compared to cells with much greater intensity. Similarly, samples with zero values for TPS may have discernable staining that simply does not exceed the threshold, is non-membranous or is localized to non-tumor cells, such as macrophages or dendritic cells. Thus, the application of thresholds and TPS in IHC poorly reflects actual PD-L1 content. Moreover, modifications of PD-L1 protein, such as N-glycosylation and prolonged storage of FFPE sections may affect detection in IHC^13,23^.

PD-L1 measurements alone provide an incomplete representation of the potential for PD-1-dependent regulation of T-cells, as PD-L2 may also drive PD-1-dependent T-cell downregulation. Although IHC assays for PD-L2 recently have been reported^25–27^, PD-L2 is seldom assessed in solid tumors. PD-L2 positive status assessed by IHC has been reported to be an independent predictor of response to pembrolizumab in head and neck squamous carcinoma^27^. Matsubara et al. reported that 44.1% of tumors from a 211 patient cohort were PD-L2 positive at a TPS threshold of >50%, whereas only 18% were PD-L1 positive at >50% TPS^25^. Moreover, they found increasing percentages of PD-L2 positive tumors as the TPS threshold was gradually reduced, such that all tumors were PD-L2 positive at the 1% threshold. The analyses by Matsubara et al. at the >50% threshold are consistent with our observation that PD-L2 abundance exceeded PD-L1 in 15 of 20 NSCLC in the subcohort we analyzed. However, their data suggest—and our data confirm—that PD-L2 abundance, like PD-L1 abundance is a continuous variable, which can be reliably measured by MS, but not by IHC.

IHC is a powerful tool for qualitative assessment of the presence and cellular localization of proteins. However, as a predominantly qualitative platform, it is not optimal for situations where a more quantitative approach is desired. Inter-observer variability of PD-L1 expression interpretation is widely acknowledged, particularly when using anything other than strict percentage cut-offs^28,29^. Lack of concordant abundance measurements generated with different PD-L1 clones combines with inter-observer variability and inherent limitations of quantitative platform to limit the utility of IHC in measuring a spectrum of protein abundance. MS provides reliable, continuous measurement of protein expression levels across the abundance range of PD-L1 and other immune checkpoints.

Four previous studies have assessed expression of IDO1 and PD-L1 proteins together in lung squamous cell carcinomas and adenocarcinomas by IHC or immunofluorescence^30–33^. IDO1 was expressed in 15% to 87%, whereas IDO1/PD-L1 co-expression ranged from 7% to 49%; the range of reported values may reflect differences in methods, reagents and scoring thresholds. In our analyses, 52% of samples contained MS-detectable PD-L1 and IDO1, whereas only 15% expressed both proteins at levels above the average for the cohort.

mRNA measurements are widely used in tissue analysis—including FFPE tissues—but our data indicate that mRNA does not reliably predict abundance of immune checkpoint proteins. Only PD-1, PD-L1 and IDO1 demonstrated correlation of protein and mRNA abundance. Even though levels of PD-1, PD-L1 and IDO1 mRNAs did correlate with their protein abundance across the sample set, mRNA does not necessarily represent the relative abundance of these proteins within individual samples. Thus, direct protein measurements are required to measure actual protein expression ratios in tissues.

TMB is an investigational biomarker associated with response to immune checkpoint inhibitors in multiple cancers^34–36^. The underlying rationale linking TMB to immunotherapy response is that higher mutation load should drive greater neoantigen diversity and more robust immune response. TMB thus might be expected to correlate with PD-L1 abundance, yet our data revealed no correlation of TMB with PD-L1 or with any of the other immune checkpoint proteins analyzed. This is consistent with the current understanding that TMB and PD-L1 abundance appear to be independent predictors of response to immune checkpoint therapeutics^36,37^.

We performed global proteome analyses of a 26 sample sub-cohort to generate deep inventories, which recently have been demonstrated to provide new understanding of how genomic abnormalities in cancer translate to phenotypes via proteins^15–18^. Here we used GSEA^38^ based on targeted measurements of PD-L1 and IDO1 to identify proteotypes associated with high expression of these drug targets. The data revealed that high PD-L1 and high IDO1 proteotypes are distinctly different. High PD-L1 abundance was associated with Myc-driven features, consistent with a previous report that high PD-L1 IHC staining correlated with Myc overexpression, primarily in squamous cell carcinomas, rather than in adenocarcinomas^39^. In our 26 sample subcohort subjected to global proteome analysis, 8 of the 13 samples with highest PD-L1 abundance were squamous cell carcinomas.

GSEA revealed a distinctly different proteotype for NSCLC expressing high levels of IDO1. IDO1 is widely understood to inhibit T-cell function by metabolizing tryptophan to kynurenine, thereby restricting availability of an essential amino acid for protein synthesis and generating a ligand for AhR-mediated induction of regulatory T-cells^40^. However, IDO1 exhibits multiple effects on immune function that appear to be independent of its catalytic activity^41^. High IDO1 expressing NSCLC displayed elevated expression of proteins mapping to interferon alpha- and gamma-responses, allograft rejection and IL6 STAT3 signaling during acute phase response. These proteotype features confirm a previously reported transcript expression pattern associated with T-cell mediated immune responses, including transplant rejection, ulcerative colitis and multiple sclerosis^42^. Interestingly, MHC presentation of peptide sequences derived from the IDO1 protein itself may contribute to immunogenicity associated with this gene expression program^43^. Distinct global proteotypes for high-PD-L1 versus high-IDO1 expressing NSCLC could merit exploration in retrospective analysis of archival FFPE specimens from completed combination trials with disappointing or ambiguous outcomes, such as the recent ECHO-202/KEYNOTE-307 phase 3 trial of pembrolizumab and epacadostat in melanoma^44^. Proteotype information may reveal previously unidentified protein pathway characteristics associated with response to drug combinations.

Our data demonstrate the feasibility of multiplexed MS measurements to quantify protein expression of immunotherapy targets and further demonstrate the superiority of MS to other technologies for quantitative protein measurements. Robust, MS-based multiplexed quantitative measurements can advance tissue protein analysis much as oligonucleotide arrays, RNA-Seq and reverse transcriptase polymerase chain reaction (RT-PCR) advanced the field of gene expression and will extend our understanding of the proteomic landscape. Although MS analyses cannot provide spatial relationships between targets and tissue cellularity, a quantitative protein data layer nevertheless provides unique information that complements other data on tissues. Most importantly, MS provides a systematic approach to quantitatively assess drug target abundance and co-expression, which will be essential to successful design and clinical evaluation of combination therapeutics.

## Methods

### Tissue specimens and immunohistochemistry

FFPE tissue blocks of primary, immune checkpoint inhibitor naïve NSCLC tissue were commercially acquired from iSpecimen (Lexington, MA, USA) and Indivumed (Frederick, MD, USA). Vendor source and tissue diagnosis are provided in Table S5. Microtomy was performed immediately prior to experiment initiation. Tissue sections were cut at 5 µm thickness and allowed to dry at room temperature overnight. IHC staining for PD-L1 was performed using the PD-L1 IHC 22C3 pharmDx kit (Agilent; Santa Clara, CA, USA) according to manufacturer instructions. Expression of PD-L1 was assessed by qualified pathologists (AH, AMG) trained and experienced in its interpretation in accordance with established guidelines.

### Nucleic acid isolation and sequencing

Four 10 µm thickness paraffin curls were obtained from each FFPE specimen. Total RNA was extracted from two curls with RNeasy FFPE (Qiagen, Germantown, MD), and quantity was assessed using a Qubit fluorometer (ThermoFisher Scientific, Waltham, MA). RT was performed using 30 ng of RNA with the SuperScript VILO cDNA Synthesis kit (ThermoFisher Scientific). cDNA libraries were prepared with the Oncomine Immune Response Research Assay and Chef-Ready Kit (ThermoFisher Scientific, Cat. No. A32928) reagents on the Ion Chef instrument (ThermoFisher Scientific). Sequencing was performed on 540 chips on the Ion Torrent S5XL (ThermoFisher Scientific). Raw FastQ files were quality- and adapter-trimmed using cutadapt (cutadapt-1.9.1) and aligned using GSNAP (v2013-11-27, command line parameters -B 5 -A sam -N 1 -t 8 -s splicesites–quality-protocol = sanger–gunzip–sam-multiple-primaries–maxsearch = 1000–npaths = 100) to build 37.p5 of the human genome. Counts were generated using a custom perl script and normalized to reads per million (RPM). For DNA extraction, QIAamp DNA FFPE Tissue Kit was used (Qiagen) with sample prepared from two curls. Quantity was assessed using a Qubit fluorometer. DNA (30 ng) was treated with uracil DNA glycosylase for 2 minutes at 37 °C and then for 10 minutes at 50 °C before input into the Oncomine Tumor Mutation Load Assay (ThermoFisher Scientific). Library preparation and templating were performed on the Ion Chef and sequencing was done on the Ion Torrent S5XL using 550 chips. Results were assessed using the associated Ion Reporter (ThermoFisher Scientific, version 5.10) workflow “w2.0 - DNA - Single Sample”. Output data are represented as mutations/Mb.

### MS analysis of FFPE specimens

Tissue sections were scraped from FFPE sections, deparaffinized, and rehydrated as described previously^23^. Protein concentration in lysates was measured using the BCA assay (ThermoFisher Scientific) and 100 μg was taken for reduction, alkylation and tryptic digestion^23^. A mixture of 50 fmol each of the stable isotope labeled peptide standards for PD-1, PD-L1, PD-L2, LAG3, IDO1, TIM3, ICOSLG, VISTA, GITR and CD40 (the target sequences are provided in Table S7). Tryptic peptide digests were fractionated by basic reverse phase liquid chromatography with disposable spin columns (Pierce High pH Reversed-Phase Peptide Fractionation Kit, Thermo Scientific, Rockford, IL, USA) according to the manufacturer’s instructions. Fractionation of labeled peptide standards spiked into a bovine albumin tryptic digest followed by MS analysis identified the fractions in which the peptides eluted. This information enabled selection of the sample fractions to analyze by targeted MS.

Targeted MS analyses were adapted from our previously reported method^23^ and were performed on an Orbitrap Fusion Lumos Tribrid instrument (Thermo Scientific, Bremen, Germany) equipped with an Easy nLC™ 1200 liquid chromatograph and a Nanospray Flex ion source (Thermo Scientific, San Jose, CA). Reverse phase liquid chromatography was done with a PepMap RSLC C18-3 micron column, 75 micron x 15 cm, eluted at 250 nL/min with a mobile phase gradient consisting of solvent A (0.1% aqueous formic acid) and solvent B [(0.1% formic acid in water/acetonitrile (1:4, v/v)]. The mobile phase was initially 5% B and then programmed to 20% B over 18 min, to 35% B over 14 min and finally to 95% B over 5 min before recycling to starting composition.

Targeted MS analysis was done by parallel reaction monitoring on the Lumos^23^. The acquisition method consisted of a full scan selected ion monitoring event followed by targeted MS2 scans as triggered by a scheduled inclusion list, with a 5 min retention time window containing the precursor *m/z* values. Retention times were determined from prior analyses of synthetic peptide standards. The MS1 scan was collected at a resolution of 30,000, an automatic gain control (AGC) target value of 5e4, and a scan range from *m/z* 350–1000. MS1 data were recorded in profile mode. The MS1 scan was followed by targeted MS2 collision induced dissociation scans at a resolution of 30,000, an AGC target value of 5e4, 1.6 *m/z* isolation window, activation Q of 0.25 and an optimized collision energy for each target of 30%. MS2 data were recorded in profile mode. Parallel reaction monitoring transitions were extracted from raw datafiles and analyzed with Skyline^45^. Peptide peak areas were calculated as the sum of the three most abundant transitions. Quantification required at least two co-eluting transitions with the correct signal intensity and with mass accuracy within 5 ppm. Unlabeled peptides with only one observed transition were assigned values of zero. Peptide abundance was calculated from the ratio of peak area for the unlabeled endogenous peptide to peak area and spike amount for the labeled internal standard.

Global proteome analyses were performed on high pH reverse phase fractionated tryptic digests of a subset of the same samples with a Lumos instrument equipped with a Waters NanoAcquity system. Peptides were loaded on a trapping column and eluted over a 75μm analytical column at 350nL min^−1^. Both columns were packed with Luna C18 resin (Phenomenex, Torrance, CA). The mass spectrometer was operated in data dependent HCD mode, with MS and MS/MS performed in the Orbitrap at 60,000 FWHM and 15,000 FWHM resolution, respectively. The instrument was run with a 3 s cycle for MS and MS/MS. The advanced peak determination algorithm was enabled. Tandem MS scans were acquired as centroided data. Peptide sequence identification from tandem mass spectra was done by database search against the human RefSeq V78 database with the MS-GF + search engine^46^. Peptide spectrum matches were filtered using IDPicker ver. 3.1^47^. Peptide spectrum matches were performed with an FDR threshold of 1% and required at least 2 distinct peptide identifications per protein identification. Spectral count data were subjected to global normalization and log transformation and differential protein abundance was represented by z-score distribution. Proteins with differential abundance as a function of PD-L1 or IDO1 measurements mapped to multiple pathways and molecular functions, as determined by GSEA against the Hallmark gene set collection of the Molecular Signatures Database^38^ using WebGestalt^48^.

## Supplementary information

Supplementary information.

Supplementary information 2.

Supplementary information 3.

Supplementary information 4.

Supplementary information 5.

## Data Availability

Global MS data are available through Proteome eXchange Accession MSV000085049 at ftp://massive.ucsd.edu/MSV000085049/. Targeted MS data are available through PanoramaWeb.org at https://panoramaweb.org/hULkB7.url.
