# Supplementary material for: Analysis of Immune Checkpoint Drug Targets and Tumor Proteotypes in Non-Small Cell Lung Cancer
Source: Sci Rep. 2020 Jun 17;10:9805. doi: 10.1038/s41598-020-66902-0 (PMC7300007; doi:10.1038/s41598-020-66902-0)
Supplement: Supplementary file 4 — Supplementary information 4. [file 41598_2020_66902_MOESM4_ESM.pdf]

# Analysis of Immune Checkpoint Drug Targets and Tumor Proteotypes in Non-Small Cell Lung Cancer

Daniel C. Liebler[1]*, Timothy R. Holzer[2], Alexander Haragan[3], Ryan D. Morrison[1], Leslie O'Neill Reising[2], Bradley L. Ackermann[2], Jeff A. Fill[2], Andrew E. Schade[2], and Aaron M. Gruver[2]*

[1]Protypia, Inc, Nashville, TN, USA

[2]Lilly Research Laboratories, Eli Lilly and Company, Indianapolis, IN, USA

[3]Institute of Translational Medicine, University of Liverpool, Liverpool, UK

**SUPPLEMENTARY FIGURES**

**Figure S1.**

**Figure S2.**

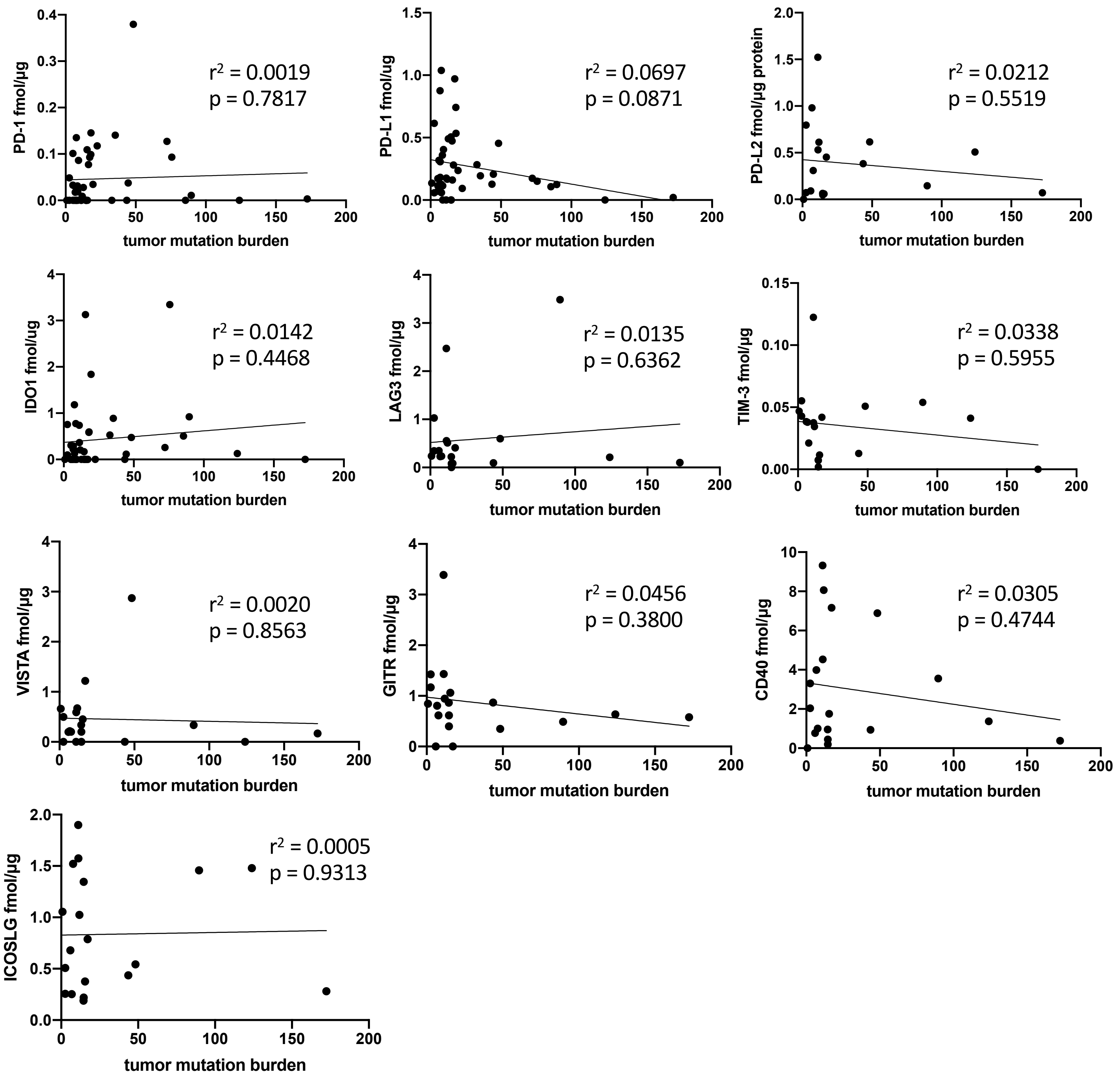


**Figure S1.** Correlation of MS protein with TMB for PD-1, PD-L1, IDO1 in the 46 sample NSCLC cohort and for PD-L2, LAG3, TIM3, ICOSLG, GITR, VISTA and CD40 in the 20 sample subcohort.

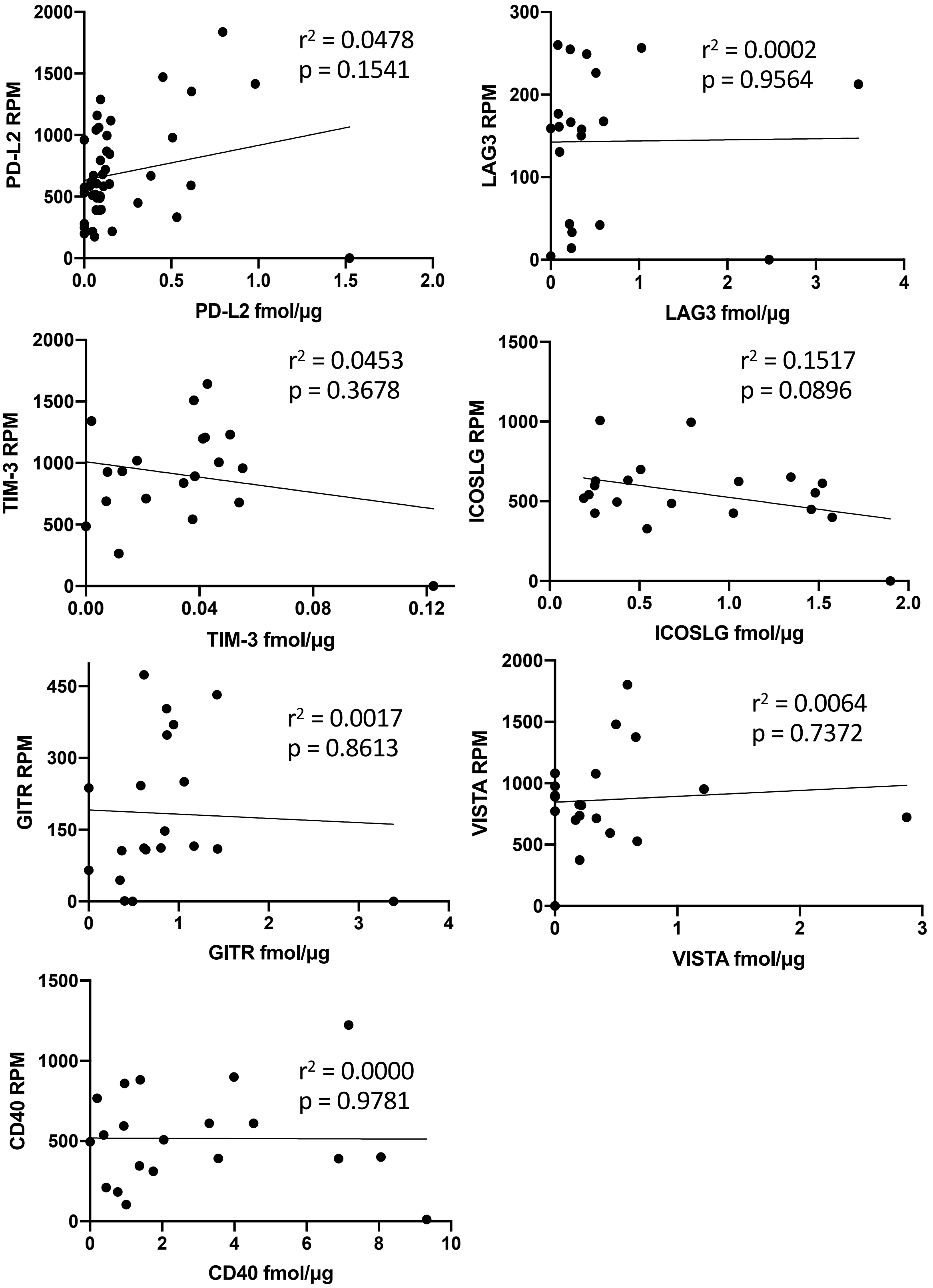


**Figure S2.** Correlation of MS protein with mRNA expression for PD-L2, LAG3, TIM3, ICOSLG, GITR, VISTA and CD40 in the 20 sample NSCLC subcohort.
